# Diabetes Pay-for-Performance Program Participation and Dialysis Risk in Relation to Educational Attainment: A Retrospective Cohort Study

**DOI:** 10.3390/healthcare11222913

**Published:** 2023-11-07

**Authors:** Yi-Shiun Tsai, Wen-Chen Tsai, Li-Ting Chiu, Pei-Tseng Kung

**Affiliations:** 1 Department of Orthopedics, Feng Yuan Hospital, Taichung 420210, Taiwan; stcomas@fyh.mohw.gov.tw; 2 Department of Health Services Administration, College of Public Health, China Medical University, Taichung 406040, Taiwan; wtsai@mail.cmu.edu.tw (W.-C.T.); litingchiu933@gmail.com (L.-T.C.); 3 Department of Healthcare Administration, Asia University, Taichung 413305, Taiwan; 4 Department of Medical Research, China Medical University Hospital, Taichung 404327, Taiwan

**Keywords:** type 2 diabetes, educational attainment, pay-for-performance, dialysis

## Abstract

Pay-for-performance (P4P) programs for diabetes care enable the provision of comprehensive and continuous health care to diabetic patients. However, patient outcomes may be affected by the patient’s educational attainment. The present retrospective cohort study aimed to examine the effects of the educational attainment of diabetic patients on participation in a P4P program in Taiwan and the risk of dialysis. The data were obtained from the National Health Insurance Research Database of Taiwan. Patients newly diagnosed with type 2 diabetes mellitus (T2DM) aged 45 years from 2002 to 2015 were enrolled and observed until the end of 2017. The effects of their educational attainment on their participation in a P4P program were examined using the Cox proportional hazards model, while the impact on their risk for dialysis was investigated using the Cox proportional hazards model. The probability of participation in the P4P program was significantly higher in subjects with a junior high school education or above than in those who were illiterate or had only attained an elementary school education. Subjects with higher educational attainment exhibited a lower risk for dialysis. Different educational levels had similar effects on reducing dialysis risk among diabetic participants in the P4P program.

## 1. Introduction

People today live in greater affluence, but their daily exercise is reduced. As a result, obesity is highly prevalent, and the incidence and prevalence of diabetes are rapidly increasing. According to the statistics of the International Diabetes Federation, there were 463 million people with diabetes worldwide in 2019, and it is estimated that the number of patients will rise to 700 million in 2045, constituting a 51% increase [1]. If diabetic patients do not control their blood glucose well, long-term hyperglycemia will lead to a series of complications, including nephropathy, retinopathy, neuropathy, cerebrovascular diseases, cardiovascular diseases, peripheral vascular diseases, ketoacidosis, hyperosmolar hyperglycemic state, and other problems [2]. Among them, about 30–46% of cases with type 2 diabetes are complicated by chronic kidney diseases; these patients ultimately require dialysis treatment [3]. The medical care burden derived from diabetes and its complications is also massive [4]. Diabetes is a chronic disease with high incidence and high medical costs; however, proper management and care can prevent or delay its complications [5,6]. Dialysis is one of the treatment strategies for end-stage renal disease (ESRD). Diabetic nephropathy is now the leading cause of ESRD in the U.S. [7]. Taiwan has the highest incidence and prevalence of dialysis globally [8], with more than 47% of dialysis cases caused by diabetes complications [9]. As the prevalence of diabetes continues to increase in Taiwan, ESRD has become a serious burden on the national healthcare system [10]. End-stage renal disease causes progressive organ damage elsewhere, leading to premature mortality. The excess mortality associated with type 2 diabetes occurs primarily in patients with chronic kidney disease [11]. Therefore, how to prevent and treat the progression of diabetic nephropathy is an important issue.

“Pay-for-performance” (P4P) is a new medical expense payment system concept aimed at improving medical care compliance through financial incentives; that is, it provides financial incentives to help hospitals and doctors provide a high-quality, low-cost, and holistic medical care model to achieve a specific medical care quality index. Pay-for-performance has been widely used in many developed countries, including the UK Quality and Outcomes Framework (QOF), the Australia Practice Incentives Program (PIP), and Taiwan’s diabetes pay-for-performance program. In Taiwan’s P4P program for diabetes, medical professionals from different disciplines constitute a shared care medical team that provides a complete care model comprising patient diagnosis, examinations, health education, and follow-up. In this system, doctors are rewarded based on the number of patients received and the status of four performance monitoring indicators, that is, the complete patient follow-up rate, which includes at least three scheduled follow-ups per year; the control rate of glycated hemoglobin; and the rate of achieving low-density lipoprotein goals [12]. P4P programs for diabetes provide appropriate care and effective use of medical resources by regularly delivering medical care. While the literature suggests that P4P programs have a limited impact on care quality [13,14], some studies suggest that P4P programs improve some process outcomes and the achievement of hemoglobin A1C, cholesterol, and blood pressure targets [15,16]. P4P programs for diabetes can reduce or delay the occurrence of complications, as well as reduce the number of times and days of hospitalization, thereby effectively controlling medical expenses [12,17,18].

People with relatively lower education are more likely to suffer from diabetes [19]. The prognosis of diabetes is found to be influenced by medical care access and quality for diabetic patients, management of blood biochemical parameters, and healthy patient behavior. The mode of medical care for diabetes has become complex. This has made preventative care more accessible to only the highly educated class of people, who find it easier to adopt appropriate and healthier behaviors [20]. Even in the literature, relatively higher education is positively correlated with better employment, higher income potential, and thus more healthcare opportunities [21]. That is, better-educated patients are less likely to suffer the adverse consequences of the disease, namely dialysis and mortality. Similarly, the research also confirms that, among diabetic patients, those with less education have poorer blood glucose control [22], and those with relatively low health literacy have a higher risk of stroke [23] and mortality [24,25]. 

While the prognosis of diabetic patients may be influenced by their educational level, patients who participate in pay-for-performance programs can receive medical care, health education, and follow-up treatment provided by professional medical teams. In this case, the subsequent influence of different educational levels on whether patients participate in a pay-for-performance program are worthy of further investigation. However, very few studies have focused on an in-depth analysis of the educational levels of diabetic patients. This study aims to compare the participation rates in a pay-for-performance program among diabetic patients with different educational levels and investigate the difference in dialysis risk among diabetic patients with varying educational levels and participation in the program.

## 2. Materials and Methods

### 2.1. Study Subjects

The main research population of this study was patients newly diagnosed with type 2 diabetes from 2002 to 2015. Data from 2000 to 2001 were taken as the washout period for newly diagnosed diabetes. These patients were subject to follow-up until the end of 2017. The study defined diabetic patients as those who had a primary or secondary diagnosis of diabetes in three outpatient visits within 365 consecutive days (ICD9: 250) or one hospitalization record and had taken diabetes-related drugs (the ATC CODE begins with A10) at least once [26,27]. Those who met the following criteria were excluded: (a) gestational diabetes, only those who sought medical care for primary and secondary diagnosis of diabetes within 270 days before delivery (ICD9: 648); (b) type 1 diabetes, defined as those who held a Major Illness/Injury Certificate for diabetes, or who had at least one outpatient diagnosis of insulin-dependent diabetes (250.x1, 250.x3) and one inpatient diagnosis of diabetic ketoacidosis (250.1x), or who had a diagnosis of insulin-dependent diabetes in at least three outpatient visits within 365 consecutive days; (c) patients who had kidney transplantation or dialysis before joining the pay-for-performance program or developing diabetes.

**Figure 1:**
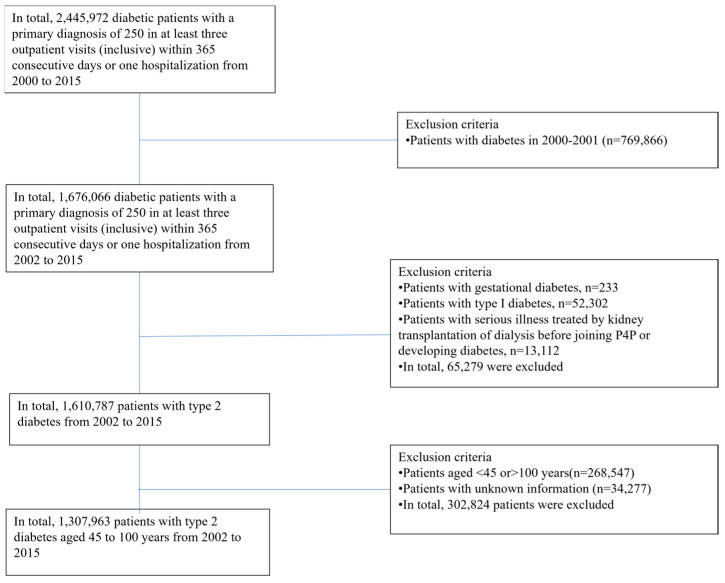
Research subject selection.

It is reasonably assumed that most people’s educational level does not change much after age 45. Thus, diabetic patients aged 45–100 years were included in the study, although patients with missing values of research variables were excluded. In total, 1,307,963 diabetic patients were included (the selection process for the research subjects is shown in Figure 1).

### 2.2. Data Sources

This is a retrospective cohort study. The data sources included the National Health Insurance Research Database and the Cause of Death File, published from 2000 to 2017, provided by the Ministry of Health and Welfare. Individuals’ educational attainment was obtained from the Household Registration Files issued by the Ministry of the Interior. 

### 2.3. Descriptions of Variables

The independent variable was educational level, which was divided into illiteracy or elementary school, junior high school, senior high school, university, and master’s degree or above, according to the educational level in the household registration document. The dependent variables were participation in the pay-for-performance program for diabetes (“yes” or “no”) and dialysis treatment (“yes” or “no”). Type 2 diabetic patients who had code “E4” in the treatment items in the outpatient medical record or code “C” as the payment category in the inpatient medical service fee calculation list were defined as participating in the program [25]. In this study, whether the patient had undergone dialysis due to chronic renal failure was based on whether dialysis was recorded in the Registry for Catastrophic Illness Patients. The Registry for Catastrophic Illness Patients includes 30 severe illnesses and injuries (e.g., chronic renal failure, malignant tumors, etc.) listed by Taiwan’s National Health Insurance Administration. Therefore, those with dialysis recorded in the Registry for Catastrophic Illness Patients were defined as dialysis patients.

The control variables included patient demographics, financial factors, environmental factors, health status, and healthcare provider characteristics. Patient demographics included gender, age, and marital status. Marital status was divided into unmarried, married, divorced, and widowed. The patient’s financial status was measured by their monthly salary, which was divided into six grades. The patients’ living area environments were divided into seven urbanization grades. Among them, Grade I indicated the highest level of urbanization, and Grade VII indicated the lowest level [28]. 

Patients’ health status was expressed by Deyo’s Charlson Comorbidity Index (CCI) and diabetes complications severity index (DCSI). The CCI modified by Deyo et al. was adopted, classifying comorbidities into 17 categories. The patients’ codes of primary and secondary diagnoses (ICD-9-CM) were converted into value weight scores to be added up to calculate CCI scores [29]. The severity of diabetic complications (diabetes complications severity index (DCSI)) was calculated by scoring the codes of primary and secondary diagnoses (ICD-9-CM) of the patients according to the presence and severity of complications and then adding the complication score values [2]. The scores of CCI and DCSI were calculated mainly based on the medical records of the research subjects in the first two years before the index date. We defined the starting point of the observation period as the index date. For patients participating in the program, the index date was the time of entering the program. For those not participating, the index date was the date of the new diagnosis of diabetes. Previous studies noted that hypertension and hyperlipidemia aggravate renal function and increase the risk of dialysis [30,31,32,33]. Therefore, “whether having hypertension” and “whether having hyperlipidemia” were added as control variables when investigating “whether having undergone dialysis”.

The characteristics of the healthcare provider were divided into two parts: diabetes treatment physician and diabetes treatment medical institution. The definition of “primary healthcare provider” varied according to whether the research subject participated in the pay-for-performance program. For patients who participated in the program, the primary healthcare providers were the doctors and institutions that helped the patient enter the program. For patients who did not participate, the institutions and doctors that the patient last visited for diabetes treatment as of the observation deadline were the primary healthcare providers. The service volume of the primary treatment physician for diabetes was calculated by the average annual service volume of the primary treatment physician for patients. According to the number of diabetic patients diagnosed and treated by the physician, the service volume of the physician was divided into low service volume (Q1), medium service volume (Q1–Q3), and high service volume (>Q3), according to quartiles for analysis [26]. The departments of primary treatment physicians for diabetes were divided into endocrinology, family medicine, internal medicine, and other departments. The primary diabetes treatment institutions were divided into four levels: medical centers, regional hospitals, local hospitals, and primary clinics. According to ownership, they were divided into public hospitals and non-public hospitals.

### 2.4. Statistical Analysis

In this study, SAS Version 9.4 (SAS Institute Inc., Cary, NC, USA) statistical software was used to perform descriptive statistics and inferential statistical analysis. All of the tests adopted two-tailed tests, and a *p*-value less than 0.05 was regarded as statistically significant. First, for descriptive statistics, whether participants participated in the program and whether dialysis occurred were presented by number, percentage, mean, and standard deviation for diabetic patients with different educational levels and control variables (including basic demographics of diabetic patients, financial factors, environmental factors, health status, and characteristics of primary healthcare providers). To explore the influence of educational level on diabetic patients’ participation in the program, a log-rank test was first used to check whether there were statistically significant differences in “whether participating in the pay-for-performance program for diabetes” among different educational levels and other independent variables.

Then, using the Cox proportional hazard model, under the control of patients’ basic demographics, financial factors, environmental factors, health status, and the characteristics of primary healthcare providers, the influence of educational level on diabetic patients’ participation in the program was investigated.

Finally, regarding the difference in the influence of educational level on the occurrence of dialysis in patients participating or not in the program, a log-rank test was used to test whether different educational levels, participation in the program, and other variables had statistical differences in the incidence of dialysis. Univariate Poisson regression was used to test the difference in the number of patients undergoing dialysis per thousand person-years among diabetic patients with different educational levels and other factors. As for whether there was any interaction between educational level and participation status in the P4P program on the risk of dialysis, we first used the Mantel-Haenszel test. Then, we used the Cox proportional hazards model to explore the influence of educational level and whether patients participated in the program on the risk of dialysis. The results are presented as the hazard ratio (HR) with a 95% confidence interval.

## 3. Results

### 3.1. Differences in Participation in the Pay-for-Performance Program among Diabetic Patients with Different Educational Levels

The study included 1,307,963 diabetic patients over a 13 year period, of whom 37.04% participated in the pay-for-performance program. Participation rates varied by educational level, as shown in Table 1. Patients with a senior high school education had the highest participation rate (40.24%), while those with an educational level of illiteracy or elementary school had the lowest participation rate (34.72%).

The Cox proportional hazards model was used to explore the differences in participation and related factors, and the study found that patients with a junior high school education or higher were more likely to participate in the program compared to those with an education level of illiteracy or elementary school. Specifically, patients with a senior high school education had a 1.13 times higher probability of participating compared to patients with an education level of illiteracy or elementary school (95% CI: 1.13–1.14). Gender, age, marital status, monthly salary, urbanization degree of residential area, severity of comorbidities, severity of diabetic complications, service volume of the primary treatment physician and department of the physician, and level and ownership type of the primary treatment medical institution also affected participation in the program (*p* < 0.05). Male gender, higher severity of comorbidities and diabetic complications, primary clinics, higher service volume of the diabetic patient’s attending physician, and the physician being an endocrinologist were associated with a higher probability of participation. Patients aged between 45 and 65 years had a higher probability of participating with increasing age, but those aged over 75 years had a decreasing probability.

### 3.2. Educational Level Associated with the Dialysis Incidence Rate (Per Thousand Person-Years) of Diabetic Patients Participating and Not Participating in the Pay-for-Performance Program

This study included new patients with type 2 diabetes from 2002 to 2015 as research subjects and followed them up until the end of 2017. The longest observation time period was 16 years, and the average observation time period was 7.6 years. The longest time to join the P4P program was 16 years, and the average time to join the P4P program was 8.8 years. We used univariate Poisson regression to compare the incidence of dialysis among diabetic patients with different educational levels. Table 2 shows that the dialysis rate during the observation period was 1.87%, and the dialysis rate per 1000 person-years was 2.46. The incidence of dialysis among diabetic patients with an educational level of illiteracy or elementary school was 2.79 per 1000 person-years—significantly higher than that among diabetic patients with an educational level of university or above (1.55). The higher the education level, the lower the dialysis rate, with a statistically significant difference (*p* < 0.05). The dialysis rate among diabetic patients in the program was relatively low, 1.75 per 1000 person-years, which was significantly lower than that among diabetic patients not in the program (the dialysis rate was 3.00 per 1000 person-years).

### 3.3. Educational Level Associated with the Risk of Dialysis in Diabetic Patients Participating and Not Participating in the Pay-for-Performance Program

**Figure 2:**
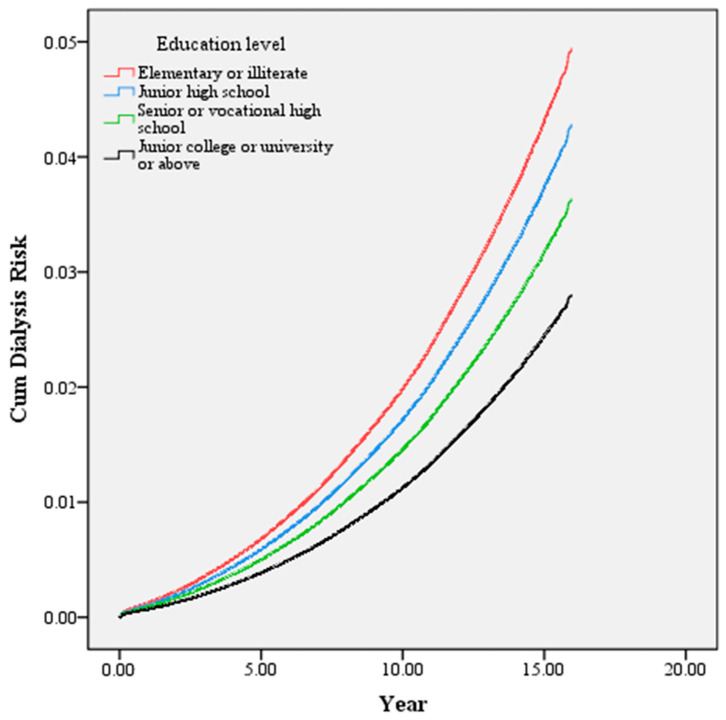
Educational level associated with the dialysis risk of diabetic patients. Subjects with higher educational attainment exhibited a lower risk for dialysis.

The study used the Cox proportional hazards model to explore the influence of educational level on the risk of dialysis in diabetic patients participating and not participating in the P4P program. After controlling for other variables, the study found that a higher educational level was associated with a lower risk of dialysis in diabetic patients (Figure 2). Compared to illiteracy or elementary school, those with a higher educational level had a lower dialysis risk (Table 3, model A), and the risk was reduced by 43% in those with an educational level of university or master’s degree (HR = 0.57, 95% CI: 0.54–0.60). Participation in the pay-for-performance program was also associated with a lower dialysis risk (HR = 0.71, 95% CI: 0.69–0.73, Table 3). There was no significant difference in the influence of participation on the dialysis status of diabetic patients with different educational levels in terms of the interaction between educational level and whether the patients participated in the program (*p* > 0.05, Table 3, model B). In addition, male gender, younger age, unmarried patients, lower monthly salary, more severe comorbidities, more severe diabetic complications, no hypertension, no hyperlipidemia, lower service volume of the primary treatment physician, medical centers, and non-public hospitals were all associated with increased dialysis risk in diabetic patients (*p* < 0.05).

## 4. Discussion

In our study, 51% of diabetic patients were illiterate or had an elementary level of education. The incidence gap between those with the highest and lowest educational levels varied by age [34]. According to the 2005 National Health Interview Survey of the Health Promotion Administration in Taiwan, the prevalence of diabetes is 18.3% among those who are illiterate, 11.4% among those with an elementary education, 4.8% among those with a junior high school education, 2.5% among those with a senior high school education, and 1.5% among those with a university degree or higher [35]. Compared with those with university degrees and above, people with lower educational levels are significantly more likely to suffer from diabetes. Most patients with diabetes in Taiwan have low education levels. Consequently, this finding is consistent with the previous literature [36,37,38]. 

This study found that the probability of participating in the program was significantly higher among diabetic patients with an educational level of junior high school, senior high school, or university or above than among those with an educational level of illiteracy or elementary school. At present, there is no research on the influence of educational level on the participation of diabetic patients in pay-for-performance programs. Nevertheless, we found that the higher the education level of a diabetic patient, the higher the participation rate. This may be because the doctors who participate in this program “potentially select” patients, that is, they may prefer those who are most likely to have better communication [39]. Diabetic patients with higher educational levels, for example, have higher health literacy, are easier to communicate with, have a better tendency toward self-care, are more likely to take preventive healthcare measures, and have a better health status [40,41,42,43]. Therefore, doctors tend to prioritize these groups in diabetes programs. Chen et al. and Kontopantelis et al. reported that the older the patient, the less likely a patient is to join such a program [44,45]. The results of this study are consistent with those of previous studies, finding the participation rate among those aged 45–55 years in the pay-for-performance program was the highest and then decreased with increasing age. This may be because fewer research subjects over 60 received a college education at the time in Taiwan. Thus, their educational levels are lower. In addition, the older the patient, the more comorbidities there will be, giving doctors a greater incentive to reverse-select [39]. This could explain why the rate of research subjects over 60 years old participating in the pay-for-performance program was low.

A total of 20–50% of diabetic patients develop diabetic nephropathy [46,47]. It takes an average of about 10–20 years for diabetic patients to develop from the first stage to the fourth stage of diabetic nephropathy and about 25 years to reach end-stage renal disease [48]. If the patient’s hyperglycemia and hypertension have not been properly controlled, the entire process can be shortened to 5 to 10 years. And 7–25% of newly diagnosed diabetic patients also have complications [49,50]. Newly diagnosed diabetes patients with complications have an increased risk of dialysis within 5 years. A previous study showed that 2.7–3.5% of type 2 diabetic patients with nephropathy progress to ESRD every year [51,52]. 

Research has found that educational level is highly correlated with the effectiveness of care for diabetic patients. The higher the educational level, the more knowledge there is about the disease, and the better the blood glucose control of diabetic patients [53,54]. Brown et al. contend that, among diabetic patients, individuals with lower socioeconomic status (family income, education, and occupation) are more likely to have poor healthcare results [55]. The results of this study showed that the higher the educational level, the lower the dialysis risk for diabetic patients. This result was consistent with the literature [56,57]. It may be that the occurrence and progression of diabetic nephropathy are not only related to the risk factors of hypertension, hyperglycemia, and urinary protein but also related to low medical knowledge of diabetic patients and insufficient access to medical care; those with low educational levels cannot effectively manage diabetes, which becomes a risk factor for diabetic nephropathy and dialysis requirements [58]. 

The results of this study showed that the diabetic patients participating in the pay-for-performance program had significantly lower dialysis risk. Pay-for-performance programs for diabetes combine multi-functional teams to provide perfect health education and care for diabetic patients. Substantial literature has proven that joining a diabetes pay-for-performance (P4P) program will improve blood sugar and glycated hemoglobin (HbA1c) levels. Hsu and Tai pointed out that diabetes patients who participate in a pay-for-performance program can effectively improve their blood sugar level [59]. Yuan et al. indicated that patients with diabetes who participate in a pay-for-performance program will improve their HbA1c level [60]. Hsieh et al. showed that joining the pay-for-performance program will reduce diabetes complications [18]. The time for joining a P4P program was far longer than in the study mentioned above; therefore, this study found that the participation of diabetic patients in the program prolonged the time it would have taken to enter the stage of terminal renal disease, thus lowering the risk of dialysis significantly compared with non-participants. This result was consistent with the literature [61,62].

In terms of the interaction between educational level and whether the patient participated in the program, the study found that the higher the educational level, the less likely the patient would need dialysis, but there was no statistically significant difference. The influence of educational level on the effectiveness of pay-for-performance (P4P) care can be divided into direct and indirect effects. Direct effects: The complexity of diabetes care (including diet, lifestyle behaviors, and medication) requires the patient’s understanding and cooperation to obtain good care outcomes. Diabetic patients with higher educational levels should better understand the diabetes medical care process and cooperate with treatment. Diabetes pay-for-performance programs comprise multifunctional healthcare teams formed by professional medical personnel, including physicians, nurses, nutritionists, and case managers. It is a complete patient-centered medical care model in which the medical team and patients cooperate with each other to provide extensive and continuous services that cover patient diagnosis, examination, health education, and patient tracking. The complete P4P medical care model will reduce the differential impact of different educational levels on dialysis risk, making the influence of educational level on the risk of dialysis similar for those staying in a P4P program. Indirect effects: Educational level affects health literacy, income, and lifestyle. Diabetic patients with higher educational levels have higher health literacy, better self-care tendencies, are more likely to take preventive health care measures, and have a better health status. Educational attainment affects income, which is essential to maintaining the quality of the complex diabetes care process. Second, low-income patients are more likely to have poor health behaviors such as smoking, physical inactivity, and bad dietary habits, thereby leading to poor outcomes in diabetes care [63]. Third, low-income patients are more likely to have multiple comorbidities and disabilities, resulting in poorer overall health [64]. Kim et al. pointed out that there is a significant interaction between receiving diabetes education courses and the educational level of people with diabetes in achieving optimal glycemic control [65]. Participation in diabetes education classes was positively associated with optimal glycemic control among patients with at least a high school education but negatively associated with optimal glycemic control among those with less than a junior high school education [65]. This is mainly because, although diabetes health education courses can improve nursing care and affect the medical outcomes of diabetes, the cognitive abilities of those receiving health education are different due to their different educational levels. However, our results are inconsistent because varying levels of education reduced the risk of dialysis among those with diabetes to a similar extent under a pay-for-performance program. This may be because P4P programs provide health education, regular follow-ups, and routine physical and biochemical examinations to provide comprehensive care, thus reducing the single impact of patients’ educational levels on health disparity.

### Strengths and Limitations

There were some limitations in our study. First, because secondary databases were used in this study, it was impossible to obtain relevant data on all patients (such as BMI, smoking status, and biochemical data such as baseline blood glucose, HbA1c level, urine albumin, and serum creatinine), which may have influenced the subsequent dialysis risk of diabetic patients. However, in this study, the presence of diabetic complications, hypertension, hyperlipidemia, and other variables were used to represent the basic health status of patients so as to reduce bias. Second, this study aimed to show the correlation between the level of education and efficacy rather than the causal relationship between the level of education and efficacy. More data may be needed to explore the causal relationship, such as collecting more detailed information on health knowledge, attitudes, health behaviors, lifestyles, eating habits, etc., through questionnaires. Third, Taiwan’s health insurance system may be different from those of other countries, so the design of the pay-for-performance program may be unique. Therefore, extrapolation of the results of this study to other countries may be limited. To the best of the authors’ knowledge, this is the first study to consider the educational level of diabetic patients and whether the patients participated in a pay-for-performance program. It is also the first to explore the differences in care effectiveness (dialysis) of diabetic patients participating in a pay-for-performance program with different educational levels. Hence, the results of this study deserve more attention. In addition, this study used the National Health Insurance Research Database to represent the national population data, which is widely understood for general medical care use. It also covers a follow-up period of more than 10 years. Thus, the results are representative.

## 5. Conclusions

This study found that the higher the educational level of diabetic patients, the better their participation status in the pay-for-performance program and the lower their dialysis risk. However, there were similar effects on reducing the dialysis risk when participating in the P4P program. Diabetic patients with lower educational levels had a higher risk of dialysis. Still, if they joined the pay-for-performance program, regardless of their educational level, they could significantly reduce the risk of dialysis, so diabetic patients with lower educational levels should join P4P programs. Lower proportions of diabetic patients with lower educational levels joined the P4P program, so patients with lower educational levels need more attention and care. These results can provide a reference for the clinical health education of diabetic patients and related healthcare policy agencies in formulating policies for diabetes health education and treatment.

## Figures and Tables

**Table 1:** Participation in the pay-for-performance program among type 2 diabetic patients with different educational levels and related factors.

				Non P4P		P4P			Adjusted Model			
Variable		N	%	n	%	n	%	*p*-Value ^a^	aHR	95% CI		*p*-Value ^b^
Total		1,307,963	100	823,551	62.96	484,412	37.04					
Educational level								<0.001				
	Illiteracy or elementary school (ref)	667,419	51.03	435,690	65.28	231,729	34.72		1.00	-	-	-
	Junior high school	255,585	19.54	154,835	60.58	100,750	39.42		1.13	1.12	1.14	<0.001
	Senior high school	266,363	20.36	159,181	59.76	107,182	40.24		1.13	1.13	1.14	<0.001
	University or above	118,596	9.07	73,845	62.27	44,751	37.73		1.05	1.03	1.06	<0.001
Gender								<0.001				
	Male (ref)	687,165	52.54	440,384	64.09	246,781	35.91		1.00	-	-	-
	Female	620,798	47.46	383,167	61.72	237,631	38.28		1.01	1.00	1.02	0.001
Age								<0.001				
	45–54 years (ref)	411,343	31.45	229,382	55.76	181,961	44.24		1.00	-	-	-
	55–64 years	425,315	32.52	252,601	59.39	172,714	40.61		1.07	1.06	1.08	<0.001
	65–74 years	285,080	21.80	189,580	66.50	95,500	33.50		1.01	1.01	1.02	0.003
	≥75 years	186,225	14.24	151,988	81.62	34,237	18.38		0.84	0.83	0.85	<0.001
Marital status								<0.001				
	Unmarried (ref)	59,175	4.52	40,158	67.86	19,017	32.14		1.00	-	-	-
	Married	957,399	73.20	584,331	61.03	373,068	38.97		1.01	1.00	1.03	0.092
	Divorced and Widowed	291,389	22.28	199,062	68.31	92,327	31.69		1.03	1.01	1.04	0.001
Monthly salary								<0.001				
	≤17,280 (ref)	304,806	23.3	201,897	66.24	102,909	33.76		1.00	-	-	-
	17,281–22,800	537,788	41.12	340,255	63.27	197,533	36.73		1.00	1.00	1.01	0.294
	22,801–28,800	99,931	7.64	59,083	59.12	40,848	40.88		1.04	1.03	1.05	<0.001
	28,801–36,300	106,987	8.18	63,945	59.77	43,042	40.23		1.06	1.05	1.07	<0.001
	36,301–45,800	131,401	10.05	78,828	59.99	52,573	40.01		1.06	1.05	1.07	<0.001
	≥45,801	127,050	9.71	79,543	62.61	47,507	37.39		0.95	0.94	0.96	<0.001
Urbanization degree of residential area								<0.001				
	1 (ref)	270,269	20.66	172,987	64.01	97,282	35.99		1.00	-	-	-
	2	349,453	26.72	216,380	61.92	133,073	38.08		1.09	1.08	1.10	<0.001
	3	256,815	19.63	164,298	63.98	92,517	36.02		1.08	1.07	1.09	<0.001
	4	228,152	17.44	138,592	60.75	89,560	39.25		1.27	1.26	1.29	<0.001
	5	46,174	3.53	30,723	66.54	15,451	33.46		1.12	1.10	1.14	<0.001
	6	80,358	6.14	52,256	65.03	28,102	34.97		1.15	1.14	1.17	<0.001
	7	76,742	5.87	48,315	62.96	28,427	37.04		1.23	1.22	1.25	<0.001
CCI								<0.001				
	0 point (ref)	764,242	58.43	449,852	58.86	314,390	41.14		1.00	-	-	-
	1 point	297,542	22.75	193,415	65.00	104,127	35.00		0.93	0.92	0.94	<0.001
	2 points	132,498	10.13	91,301	68.91	41,197	31.09		0.91	0.90	0.92	<0.001
	≥3 points	113,681	8.69	88,983	78.27	24,698	21.73		0.83	0.82	0.85	<0.001
DCSI								<0.001				
	0 point (ref)	924,481	70.68	577,929	62.51	346,552	37.49		1.00	-	-	-
	1 point	183,817	14.05	102,490	55.76	81,327	44.24		1.27	1.26	1.28	<0.001
	≥2 points	199,665	15.27	143,132	71.69	56,533	28.31		1.16	1.15	1.17	<0.001
Service volume of primary treatment physician								<0.001				
	Low (ref)	327,528	25.04	304,892	93.09	22,636	6.91		1.00	-	-	-
	Medium	653,521	49.96	441,950	67.63	211,571	32.37		4.65	4.59	4.72	<0.001
	High	326,914	24.99	76,709	23.46	250,205	76.54		8.50	8.37	8.63	<0.001
Department of primary treatment physician								<0.001				
	Endocrinology	314,182	24.02	71,164	22.65	243,018	77.35		3.70	3.66	3.75	<0.001
	Family medicine	261,953	20.03	169,829	64.83	92,124	35.17		2.40	2.38	2.43	<0.001
	Internal medicine	256,494	19.61	167,565	65.33	88,929	34.67		2.18	2.15	2.20	<0.001
	Other departments (ref)	475,334	36.34	414,993	87.31	60,341	12.69		1.00	-	-	-
Level of primary treatment medical institution								<0.001				
	Medical center (ref)	249,709	19.09	172,770	69.19	76,939	30.81		1.00	-	-	-
	Regional hospital	320,825	24.53	205,177	63.95	115,648	36.05		1.20	1.19	1.21	<0.001
	Local hospital	207,602	15.87	129,958	62.60	77,644	37.40		1.43	1.42	1.45	<0.001
	Primary clinic	529,827	40.51	315,646	59.58	214,181	40.42		1.65	1.63	1.66	<0.001
Ownership of primary treatment medical institution								<0.001				
	Public (ref)	294,221	22.49	173,873	59.10	120,348	40.90		1.00	-	-	-
	Non-public	1,013,742	77.51	649,678	64.09	364,064	35.91		0.88	0.87	0.88	<0.001

Note: ^a^: the results of Log-rank test analysis; ^b^: the results of Cox proportional hazard model.

**Table 2:** Educational attainment and related factors associated with the occurrence of dialysis in patients with type 2 diabetes.

				No Dialysis		Dialysis					
Variable		N	%	n	%	n	%	*p*-Value ^a^	Observed Person-Years	Dialysis Rate Per Thousand Person-Years	*p*-Value ^b^
Total		1,307,963	100	1,283,509	98.13	24,454	1.87		9,926,679	2.46	
Educational level								<0.001			
	Illiteracy or elementary school (ref)	667,419	51.03	652,890	97.82	14,529	2.18		5,216,353	2.79	-
	Junior high school	255,585	19.54	250,975	98.20	4610	1.80		1,880,218	2.45	<0.001
	Senior high school	266,363	20.36	262,399	98.51	3964	1.49		1,957,429	2.03	<0.001
	University or above	118,596	9.07	117,245	98.86	1351	1.14		872,679	1.55	<0.001
P4P								<0.001			
	Not participating (ref)	823,551	62.96	806,537	97.93	17,014	2.07		5,672,503	3.00	-
											
	Participating	484,412	37.04	476,972	98.46	7440	1.54		4,254,176	1.75	<0.001
Gender								<0.001			
	Male	687,165	52.54	672,880	97.92	14,285	2.08		5,068,670	2.82	-
	Female	620,798	47.46	610,629	98.36	10,169	1.64		4,858,010	2.09	<0.001
Age								<0.001			
	45–54 years	411,343	31.45	402,659	97.89	8684	2.11		3,472,436	2.50	-
	55–64 years	425,315	32.52	418,103	98.30	7212	1.70		3,320,146	2.17	<0.001
	65–74 years	285,080	21.80	279,536	98.06	5544	1.94		2,147,769	2.58	0.066
	≥75 years	186,225	14.24	183,211	98.38	3014	1.62		986,327.3	3.06	<0.001
											
Marital status								<0.001			
	Unmarried	59,175	4.52	57,784	97.65	1391	2.35		386,415.6	3.60	-
	Married	957,399	73.20	939,706	98.15	17,693	1.85		7,559,209	2.34	<0.001
	Divorced and widowed	291,389	22.28	286,019	98.16	5370	1.84		1,981,055	2.71	<0.001
Monthly salary								<0.001			
	≤17,280	304,806	23.3	297,759	97.69	7047	2.31		2,256,063	3.12	-
	17,281–22,800	537,788	41.12	527,327	98.05	10,461	1.95		4,121,267	2.54	<0.001
	22,801–28,800	99,931	7.64	98,194	98.26	1737	1.74		798,308.7	2.18	<0.001
	28,801–36,300	106,987	8.18	105,350	98.47	1637	1.53		796,703.4	2.05	<0.001
	36,301–45,800	131,401	10.05	129,524	98.57	1877	1.43		973,800.3	1.93	<0.001
	≥45,801	127,050	9.71	125,355	98.67	1695	1.33		980,537.1	1.73	<0.001
Urbanization degree of residential area								<0.001			
	1	270,269	20.66	265,215	98.13	5054	1.87		2,119,262	2.38	-
	2	349,453	26.72	342,999	98.15	6454	1.85		2,681,828	2.41	0.629
	3	256,815	19.63	251,831	98.06	4984	1.94		1,931,752	2.58	<0.001
	4	228,152	17.44	223,900	98.14	4252	1.86		1,700,622	2.50	0.023
	5	46,174	3.53	45,376	98.27	798	1.73		340,588.6	2.34	0.643
	6	80,358	6.14	78,864	98.14	1494	1.86		590,833.1	2.53	0.047
	7	76,742	5.87	75,324	98.15	1418	1.85		561,793.4	2.52	0.073
CCI								<0.001			
	0 point	764,242	58.43	750,564	98.21	13,678	1.79		6,106,230	2.24	-
	1 point	297,542	22.75	293,653	98.69	3889	1.31		2,297,878	1.69	<0.001
	2 points	132,498	10.13	129,274	97.57	3224	2.43		917,316.6	3.51	<0.001
	≥3 points	113,681	8.69	110,018	96.78	3663	3.22		605,254.8	6.05	<0.001
DCSI								<0.001			
	0 point	924,481	70.68	914,023	98.87	10,458	1.13		7,309,283	1.43	-
	1 point	183,817	14.05	180,906	98.42	2911	1.58		1,423,628	2.04	<0.001
	≥2 points	199,665	15.27	188,580	94.45	11,085	5.55		1,193,769	9.29	<0.001
Hypertension											
	No	634,640	48.52	622,741	98.13	11,899	1.87		5,035,531	2.36	-
	Yes	673,323	51.48	660,768	98.14	12,555	1.86		4,891,149	2.57	<0.001
Hyperlipemia								<0.001			
	No	1,242,365	94.98	1,218,719	98.10	23,646	1.90		9,440,726	2.50	-
	Yes	65,598	5.02	64,790	98.77	808	1.23		485,952.9	1.66	<0.001
Service volume of primary treatment physician								<0.001			
	Low	327,528	25.04	320,713	97.92	6815	2.08		2,227,758	3.06	-
	Medium	653,521	49.96	640,841	98.06	12,680	1.94		4,927,195	2.57	<0.001
	High	326,914	24.99	321,955	98.48	4959	1.52		2,771,726	1.79	<0.001
Level of primary treatment medical institution								<0.001			
	Medical center	249,709	19.09	243,274	97.42	6435	2.58		1,880,848	3.42	-
	Regional hospital	320,825	24.53	313,008	97.56	7817	2.44		2,454,255	3.19	<0.001
	Local hospital	207,602	15.87	203,346	97.95	4256	2.05		1,571,450	2.71	<0.001
	Primary clinic	529,827	40.51	523,881	98.88	5946	1.12		4,020,127	1.48	<0.001
Ownership of primary treatment medical institution								<0.001			
	Public	294,221	22.49	288,375	98.01	5846	1.99		2,282,277	2.56	-
	Non-public	1,013,742	77.51	995,134	98.16	18,608	1.84		7,644,402	2.43	0.001

Note: ^a^: log-rank test results; ^b^: the univariate Poisson regression analysis results.

**Table 3:** Dialysis risk among type 2 diabetes with different educational attainment participating and not participating in the pay-for-performance program and related factors.

		Adjusted Model A				Adjusted Model B			
Variable		aHR	95% CI		*p*-Value	aHR	95% CI		*p*-Value
Educational level									
	Illiteracy or elementary school (ref)	1.00	-	-	-	1.00	-	-	-
	Junior high school	0.87	0.84	0.90	<0.001	0.87	0.83	0.91	<0.001
	Senior high school	0.74	0.71	0.77	<0.001	0.75	0.72	0.78	<0.001
	University or above	0.57	0.54	0.60	<0.001	0.59	0.55	0.63	<0.001
P4P									
	Not participating (ref)	1.00	-	-	-	1.00	-	-	-
	Participating	0.71	0.69	0.73	<0.001	0.72	0.69	0.75	<0.001
Interaction									
	P4P × Illiteracy or elementary school (ref)					1.00	-	-	-
	P4P × Junior high school					1.00	0.93	1.07	0.979
	P4P × Senior high school					0.96	0.89	1.03	0.246
	P4P × University or above					0.88	0.78	1.00	0.054
Gender									
	Male (ref)	1.00	-	-	-	1.00	-	-	-
	Female	0.75	0.73	0.77	<0.001	0.75	0.73	0.77	<0.001
Age									
	45–54 years (ref)	1.00	-	-	-	1.00	-	-	-
	55–64 years	0.77	0.75	0.80	<0.001	0.77	0.75	0.80	<0.001
	65–74 years	0.73	0.70	0.76	<0.001	0.73	0.71	0.76	<0.001
	≥75 years	0.69	0.66	0.72	<0.001	0.69	0.66	0.72	<0.001
Marital status									
	Unmarried (ref)	1.00	-	-	-	1.00	-	-	-
	Married	0.78	0.74	0.82	<0.001	0.78	0.74	0.82	<0.001
	Divorce and widowed	0.89	0.84	0.94	<0.001	0.89	0.84	0.94	<0.001
Monthly salary									
	≤17,280 (ref)	1.00	-	-	-	1.00	-	-	-
	17,281–22,800	0.86	0.83	0.89	<0.001	0.86	0.83	0.89	<0.001
	22,801–28,800	0.82	0.77	0.86	<0.001	0.82	0.77	0.86	<0.001
	28,801–36,300	0.80	0.76	0.85	<0.001	0.80	0.76	0.85	<0.001
	36,301–45,800	0.75	0.71	0.79	<0.001	0.75	0.71	0.79	<0.001
	≥45,801	0.71	0.67	0.75	<0.001	0.71	0.67	0.75	<0.001
Urbanization degree of residential area									
	1 (ref)	1.00	-	-	-	1.00	-	-	-
	2	1.04	1.00	1.08	0.041	1.04	1.00	1.08	0.038
	3	1.08	1.04	1.12	<0.001	1.08	1.04	1.12	<0.001
	4	1.05	1.01	1.10	0.021	1.05	1.01	1.10	0.019
	5	0.92	0.85	1.00	0.036	0.92	0.85	1.00	0.037
	6	1.02	0.96	1.08	0.587	1.02	0.96	1.08	0.573
	7	1.02	0.96	1.08	0.603	1.02	0.96	1.08	0.590
CCI									
	0 point (ref)	1.00	-	-	-	1.00	-	-	-
	1 point	0.54	0.52	0.56	<0.001	0.54	0.52	0.56	<0.001
	2 points	1.02	0.98	1.06	0.354	1.02	0.98	1.06	0.352
	≥3 points	1.33	1.27	1.38	<0.001	1.33	1.27	1.38	<0.001
DCSI									
	0 point (ref)	1.00	-	-	-	1.00	-	-	-
	1 point	1.48	1.42	1.54	<0.001	1.48	1.42	1.54	<0.001
	≥2 points	6.24	6.06	6.42	<0.001	6.24	6.06	6.43	<0.001
Hypertension									
	No (ref)	1.00	-	-	-	1.00	-	-	-
	Yes	0.93	0.91	0.96	<0.001	0.93	0.91	0.96	<0.001
Hyperlipemia									
	No (ref)	1.00	-	-	-	1.00	-	-	-
	Yes	0.67	0.62	0.72	<0.001	0.67	0.62	0.72	<0.001
Service volume of primary treatment physician									
	Low (ref)	1.00	-	-	-	1.00	-	-	-
	Medium	0.88	0.85	0.90	<0.001	0.88	0.85	0.90	<0.001
	High	0.64	0.61	0.67	<0.001	0.64	0.61	0.67	<0.001
Level of primary treatment medical institution									
	Medical center	1.00	-	-	-	1.00	-	-	-
	Regional hospital	0.95	0.92	0.99	0.005	0.95	0.92	0.99	0.005
	Local hospital	0.79	0.76	0.82	<0.001	0.79	0.76	0.82	<0.001
	Primary clinic	0.49	0.47	0.50	<0.001	0.49	0.47	0.50	<0.001
Ownership of primary treatment medical institution									
	Public (ref)	1.00	-	-	-	1.00	-	-	-
	Non-public	1.05	1.02	1.08	0.001	1.05	1.02	1.08	0.001

## Data Availability

Data are available from the Health and Welfare Data Science Center of the Ministry of Health and Welfare (MOHW) (https://www.mohw.gov.tw/mp-2.html (accessed on 1 May 2023)), Taiwan. All interested researchers can apply to use the database managed by the MOHW. Due to legal restrictions imposed by the Taiwanese government related to the Personal Information Protection Act, the database cannot be made publicly available. Raw data from the Health and Welfare Data Science Center cannot be obtained. These restrictions prohibit the authors from making the minimal dataset publicly available.
